# A serum metabolomics study of vascular cognitive impairment patients based on Traditional Chinese medicine syndrome differentiation

**DOI:** 10.3389/fmolb.2023.1305439

**Published:** 2023-12-05

**Authors:** Li Liu, Yi-fei Qi, Min Wang, Bao-xin Chen, Qing-bing Zhou, Wen-xin Tong, Ying Zhang

**Affiliations:** ^1^ Graduate School, China Academy of Chinese Medical Sciences, Beijing, China; ^2^ Institute of Geriatric Medicine, Xiyuan Hospital, China Academy of Chinese Medical Sciences, Beijing, China; ^3^ Beijing University of Chinese Medicine, Beijing, China; ^4^ Second Department of Encephalopathy, Dongfang Hospital, Beijing University of Chinese Medicine, Beijing, China

**Keywords:** vascular cognitive impairment (VCI), kidney-yang deficiency syndrome (KYDS), Traditional Chinese medicine (TCM), metabolomics, glycerophospholipids, lipids

## Abstract

**Objective:** Vascular cognitive impairment (VCI) accounts for approximately 50%–70% of all dementia cases and poses a significant burden on existing medical systems. Identifying an optimal strategy for preventing VCI and developing efficient symptomatic treatments remains a significant challenge. Syndrome differentiation represents a fundamental approach for personalized diagnosis and treatment in Traditional Chinese Medicine (TCM) and aligns with the principles of precision medicine. The objective of this study was to elucidate the metabolic characteristics of VCI based on TCM syndrome differentiation, thus providing novel insights into the diagnosis and treatment of VCI.

**Methods:** A 2-year cross-sectional cognitive survey was conducted in four communities in Beijing between September 2020 and November 2022. The syndrome differentiation of participants was based on the Kidney-Yang Deficiency Syndrome Scale (KYDSS), which was originally developed by Delphi expert consultation. The identification of serum metabolites was performed by Ultra performance liquid chromatography (UPLC) analysis coupled with an electrospray ionization quadruple time-of-flight mass spectrometer (ESI-QTOF MS). Multivariate, univariate, and pathway analyses were used to investigate metabolic changes. Logistic regression models were also used to construct metabolite panels that were capable of discerning distinct groups. Phospholipase A2 (PLA2) levels were measured by a commercial ELISA kit.

**Results:** A total of 2,337 residents completed the survey, and the prevalence of VCI was 9.84%. Of the patients with VCI, those with Kidney-Yang deficiency syndrome (VCIS) accounted for 70.87% of cases and exhibited more severe cognitive impairments. A total of 80 participants were included in metabolomics study, including 30 with VCIS, 20 without Kidney-Yang deficiency syndrome (VCINS), and 30 healthy control participants (C). Ultimately, 45 differential metabolites were identified when comparing the VCIS group with group C, 65 differential metabolites between the VCINS group and group C, and 27 differential metabolites between the VCIS group and the VCINS group. The downregulation of phosphatidylethanolamine (PE), and phosphatidylcholine (PC) along with the upregulation of lysophosphatidylethanolamine (LPE), lysophosphatidylcholine (LPC), phosphatidic acid (PA) and phospholipase A2 (PLA2) can be considered as the general metabolic characteristics associated with VCI. Dysfunction of glycerophospholipids, particularly LPEs and PCs, was identified as a key metabolic characteristic of VCIS. In particular Glycerophospho-N-Arachidonoyl Ethanolamine (GP-NArE) was discovered for the first time in VCI patients and is considered to represent a potential biomarker for VCIS. The upregulation of PLA2 expression was implicated in the induction of alterations in glycerophospholipid metabolism in both VCIS and VCINS. Moreover, robust diagnostic models were established based on these metabolites, achieving high AUC values of 0.9322, 0.9550, and 0.9450, respectively.

**Conclusion:** These findings contribute valuable information relating to the intricate relationship between metabolic disorders in VCI, neurodegeneration and vascular/neuroinflammation. Our findings also provide a TCM perspective for the precise diagnosis and treatment of VCI in the context of precision medicine.

## 1 Introduction

Vascular cognitive impairment (VCI) refers to the entire spectrum of vascular brain pathologies that contribute to any degree of cognitive impairment, ranging from subjective cognitive decline to dementia, including subjective cognitive impairment, mild cognitive impairment (MCI) and vascular dementia (VD) (Van Der Flier et al., 2018). As life span increases, the burden of cognitive impairment on patients, families, healthcare, and long-term care systems is growing and becoming a major concern (Dichgans and Leys, 2017). In China, the prevalence of mild cognitive impairment (MCI) has been reported to be 20.8%; etiological classification has demonstrated that vascular-related MCI is the most common subtype (42.0%) (Jia et al., 2014). Therefore, as society ages, VCI will increasingly emerge as a pathological condition that can exert significant impact on overall well being and quality-of-life. However, thus far, no previous studies have focused on the identification of specific markers and the development of disease-modifying pharmacological therapeutics; thus VCI remains a significant global challenge. Therefore, it is critical that we identify an optimal strategy for the prevention of VCI and develop efficient symptomatic treatments.

Traditional Chinese medicine (TCM) syndrome is the generalization of disease states based on the comprehensive analysis of clinical information gained by the four main diagnostic procedures of TCM (observation, listening, questioning, and pulse analysis), and it is used to guide the choice of treatment (Jiang et al., 2012). Syndrome differentiation, as a fundamental approach in TCM diagnosis and treatment, discerns the functional states of an individual’s physiology and pathology while encompassing the concept of personalized treatment, thereby aligning with the principles of precision medicine. In accordance with the principles of TCM theory, the kidneys serve as the foundation for congenital constitution and regulate development and metabolism in the human body. In addition, the kidneys are interconnected with the brain *via* meridians, thereby influencing cognitive functionality at the physiological level. Kidney-Yang Deficiency Syndrome (KYDS), as one of the most common clinical syndromes, is characterized by warm dysfunction and metabolic disorder of body fluid. This condition results in aversion to coldness in the waist and limbs, aches in the waist and knees, tinnitus, nocturia, psychasthenia and fifth-watch diarrhea (Lu et al., 2011). Recent research reported that inhibition of the hypothalamic-pituitary-thyroid/gonadal axis (HPT axis) and hypothalamic-pituitary-adrenal axis (HPA axis) may present the pathological mechanism of KYDS (Lu et al., 2011; Chen et al., 2018), thus corresponding with the kidney-brain theory of TCM.

It is also important to note that KYDS is detectable in a particularly high proportion of patients with VCI (Fu et al., 2017). This perspective was supported by a 2-year cross-sectional cognitive survey conducted prior to a metabolomics investigation which also provided evidence for the selection of TCM syndrome in this study. In this cross-sectional survey, we utilized the Kidney-Yang Deficiency Syndrome Scale (KYDSS) (Supplementary Material S1) which was originally developed by Delphi expert consultation involving 30 TCM experts; this included 2,337 residents from four communities in Haidian District. Our findings revealed that VCI with Kidney-Yang deficiency syndrome (VCIS) accounted for 70.87% of all cases and was associated with more severe cognitive impairments. Given the intricate interplay between the kidneys, metabolism and cognitive function, along with the clinical features of VCIS, we hypothesized that VCIS represents a pivotal syndrome in the progression of VCI.

Based on the high-throughput identification and quantification of small molecule metabolites in cells, tissues, and biofluids, metabolomics serves as a powerful tool for mapping global biochemical changes and the discovery of new biomarkers for disease (German et al., 2005). In addition, bioinformatic metabolic pathway and network analysis can be used to reduce the complexity of the data (Rinschen et al., 2019), while liquid chromatography-high resolution mass spectrometry (LC-MS) represents a powerful approach to identify metabolites that are biologically important (Chen et al., 2021). Utilizing metabolomics as the primary methodology, this study aimed to achieve several objectives: firstly, to provide a comprehensive overview of the general metabolic characteristics of VCI; secondly, with VCINS and healthy control participants (C) serving as control groups, to investigate the specific metabolic features of VCIS; and finally, to develop a robust diagnostic model based on metabolites for accurate differentiation between the three groups of patients. By integrating traditional Chinese medicine syndrome differentiation and precision medicine approaches, the findings of our study provide novel insights for assessing the severity of cognitive impairment and facilitating personalized or precision medicine interventions for VCI.

## 2 Materials and methods

### 2.1 Clinical cohort

A total of 2,337 residents were surveyed in Beijing from September 2020 to November 2022, ultimately 80 participants who met the inclusion and exclusion criteria were recruited, including 30 vascular cognitive impairment patients with Kidney-Yang Deficiency syndrome (VCIS), 20 of non-vascular cognitive impairment patients without Kidney-Yang Deficiency syndrome (VCINS) and 30 of healthy control participants (C), and their blood samples were collected for metabolic study.

The diagnosis of VCI was based on Guidelines for Diagnosis and Treatment of Vascular Cognitive Impairment in China 2019 (Cognitive Impairment Professional Committee NB, Chinese Medical Doctor Association, 2019) and Progress toward standardized diagnosis of vascular cognitive impairment: Guidelines from the Vascular Impairment of Cognition Classification Consensus Study (Skrobot et al., 2018). Ascertain Dementia 8 questionnaire (AD8) And Montreal Cognitive Assessment (MoCA) Beijing version were used to diagnosis and access cognitive impairment. The diagnosis of Kidney-Yang Deficiency was based on the Kidney-Yang Deficiency Syndrome Scale (KYDSS) Supplementary Material S1, which was developed by 30 TCM experts using Delphi expert consultation. The scale included 10 core items, and the score was completed by physicians after consultation with patients. The total score >8 was diagnosed as Kidney-Yang Deficiency Syndrome. The appearance of tongue and pulse was used as auxiliary diagnosis.

The inclusion criteria were as follows: 1) aged 40–80 years old, male or female; 2) complained of or were reported by a relative or accompanying person as having a decline in cognitive function; 3) obtained the following results in a neuropsychological assessment under professional instruction: AD8 score≥2, MoCA score <26 points; 4) had imaging evidence that supports cerebrovascular disease, with or without a medical history of transient ischemic attack or other types of stroke; 5) had signs and symptoms caused by cerebrovascular disease (e.g., hemiplegia, hemiparesis, problems with understanding or forming speech, numbness or strange sensations). 6) diagnosed as Kidney-Yang Deficiency Syndrome or non-Kidney-Yang Deficiency Syndrome. The exclusion criteria were as follows: 1) had co-morbidity of AD, Parkinson’s disease, frontotemporal dementia, Huntington’s disease, demyelinating disease, post-traumatic dementia, or central nervous system infection; 2) had epilepsy, psychosis or depression; 3) had clinically significant gastrointestinal, renal, hepatic, respiratory, or other systemic diseases; and 4) had severe visual or hearing impairment, severe aphasia or limb dysfunction which might affect the assessment. 5) had participated in other drug clinical trials within 3 months. Healthy individuals without VCI, diabetes, cardiovascular disease, stroke, hyperlipidemia and other medical histories who was judged as non-Kidney-Yang Deficiency Syndrome were screened as the C group.

The study followed the ethical principles of human medical research in the Declaration of Helsinki. The research protocol was approved by the Ethics Committee of Xiyuan Hospital, China Academy of Chinese Medical Sciences (No. 2021XLA001-1), and written informed consent was obtained from each subject.

### 2.2 Sample collection and clinical measures

The researchers collected information of medical history, living and eating habits and assisted participants to complete the 4 scales (AD8, MoCA, depression and KYDSS).

5 mL of fasting morning blood samples was collected from the median cubital vein and placed in a precooling EDTA (Ethylene diamine tetraacetic acid) anticoagulation tube. Blood samples were centrifuged at 4,000 r/min at 4°C for 10 min after blood collection. The separated plasma samples were put into liquid nitrogen and transferred to −80°C before further use. No repeated freezing and thawing occurred before sample processing to avoid potential degradation risks of metabolites. Measurements of body mass index (BMI), systolic blood pressure (SBP), diastolic blood pressure (DBP) were recorded. Fasting blood-glucose (FPG), Glycosylated hemoglobin (HbA1c), Homocysteine (Hcy), Triglyceride (TG), Total cholesterol (TC), Low-density lipoprotein (LDL-C), and High-density lipoprotein (HDL-C) were measured immediately.

### 2.3 Sample preparation

A total of 50 μL of each plasma sample was transferred to a single well in a 96-well sample collection plate, and 150 μL of acetonitrile containing internal standard was added to each plasma sample. After mixing for 3min, the plate was transferred to −20°C (overnight). After centrifugation at 4,680 rpm for 15min at 4°C, and the all supernatant was transferred to a new single well in a 96-well sample collection plate. Then, the plate was transferred to 2°C–8°C (10 h). After centrifugation at 4,680 rpm for 15min at 4°C, and the all supernatant was transferred to a single well in a 96-well sample collection plate. And the plate was used for UPLC-MS/MS analysis.

### 2.4 Metabolomics analysis by electrospray-ionization quadrupole time-of-flight mass spectrometry (ESI-QTOF MS)

Quality control (QC) samples were obtained by mixing 20 μL of the supernatant of each sample and vortexing for 60 s. Samples for metabolomics analysis were prepared by pooling aliquots from all samples. QC samples were injected every six samples during data acquisition. Ultrahigh-performance liquid chromatography equipped with quadrupole time-of-flight mass spectrometry (UPLC–QTOF-MS) analyses were performed using a UPLC system (ACQUITY UPLC *I*-Class, Waters) coupled to an electrospray ionization quadruple time-of-flight mass spectrometer (ESI-QTOF MS) (SYNAPT G2-S HDMS, Waters). A Waters ACQUITY HSS T3 column [particle size, 1.8 μm; 100 mm (length) x 2.1 mm (i.d.)] was used for liquid chromatography (LC) separation and the column temperature was kept at 40°C. The flow rate was 0.4 mL/min and the sample injection volume was 5 μL. The mobile phase A was 0.1% FA in water, and mobile phase B was 0.1% FA in ACN. The linear gradient was set as follows: initial to 0.2 min: 2% B, 0.2–4.0 min 2% B to 60% B, 4.0–5.0 min: 60% B to 60% B, 5.0–9.0 min: 60% B to 95% B, 9.0–10.5 min: 95% B, 10.6–13.0 min: 2% B. High-accuracy MS data were acquired by MassLynx 4.1 software (Waters). The capillary voltage was 3.0 kV for positive mode and 2.5 kV for negative mode; the cone voltage was 30 V for both modes. The source temperature was set at 120°C with a cone gas flow of 150 L/h. The desolvation temperature was set at 550°C with a desolvation gas flow of 1000 L/h. Leucine-enkephalin (Waters) was used as the lock mass generating a reference ion at m/z 556.2771 in positive mode and m/z 554.2615 in negative mode; this was introduced by a lockspray at 10 μL/min for data calibration. The MS^E^ data were acquired in centroid mode using ramp collision energy in two scan functions. For low energy mode, we used a scan range of 50–1,200 Da, a scan time of 0.15 s, and a collision energy of 4 V. For high energy mode, we used a scan range of 50–1,200 Da, a scan time of 0.15 s, and a collision energy ramp of 20–35 V.

### 2.5 Data processing

Raw metabolomics data were imported to commercial software Progenesis QI (Version 2.4, Waters, United States) for data processing, which included peak picking, peak alignment and acquiring compound-associated information such as m/z, retention time and intensity. The annotation of metabolites is carried out through HMDB database. Next, data filtering was performed to delete low-quality data. Ions with a relative standard deviation (RSD) of more than 30% in quality control (QC) samples were filtered. These filtered ions fluctuated too much among samples and were not included for further analysis. (O)PLS-DA (Orthogonal signal correction partial least square discrimination analysis) was performed and VIP (variable importance in projection) was calculated by MetaboAnalyst 5.0 (https://www.metaboanalyst.ca/) and R project. Pathway analysis was performed by MetaboAnalyst 5.0. Figure 6C was drawn by Figdraw (www.figdraw.com).

### 2.6 PLA2 measurement by ELISA kits

The PLA2 contents were measured using ELISA kits (Jiangsu Jingmei Biological Technology Co., Ltd, China) following the manufacturer’s instructions. The assays were conducted using MD microplate readers (Molecular Devices, United States) for accurate measurements.

### 2.7 Statistical analysis

Comparisons were performed with T-test, Wilcoxon–Mann–Whitney, and one-way ANOVA as appropriate. Non-parametric tests were used for comparing ordinal or non-HC variables. A two-tailed Student’s t-test was further used to verify whether the metabolites of difference between groups were significant. Unique metabolites were selected with VIP> 1.0. Significance was defined as *p* < 0.05. The metabolites exhibiting *p* < 0.05 and VIP >1 in each comparison were identified as differential metabolites. Logistic regression models were constructed using the GLM function in R language. The area under the curve (AUC) value was calculated for each differential metabolite, and those with AUC >0.7 were selected and combined to form a model. Subsequently, a new model was constructed to calculate the AUC value again.

## 3 Results

### 3.1 Demographic and clinical characteristics

**FIGURE 1:**
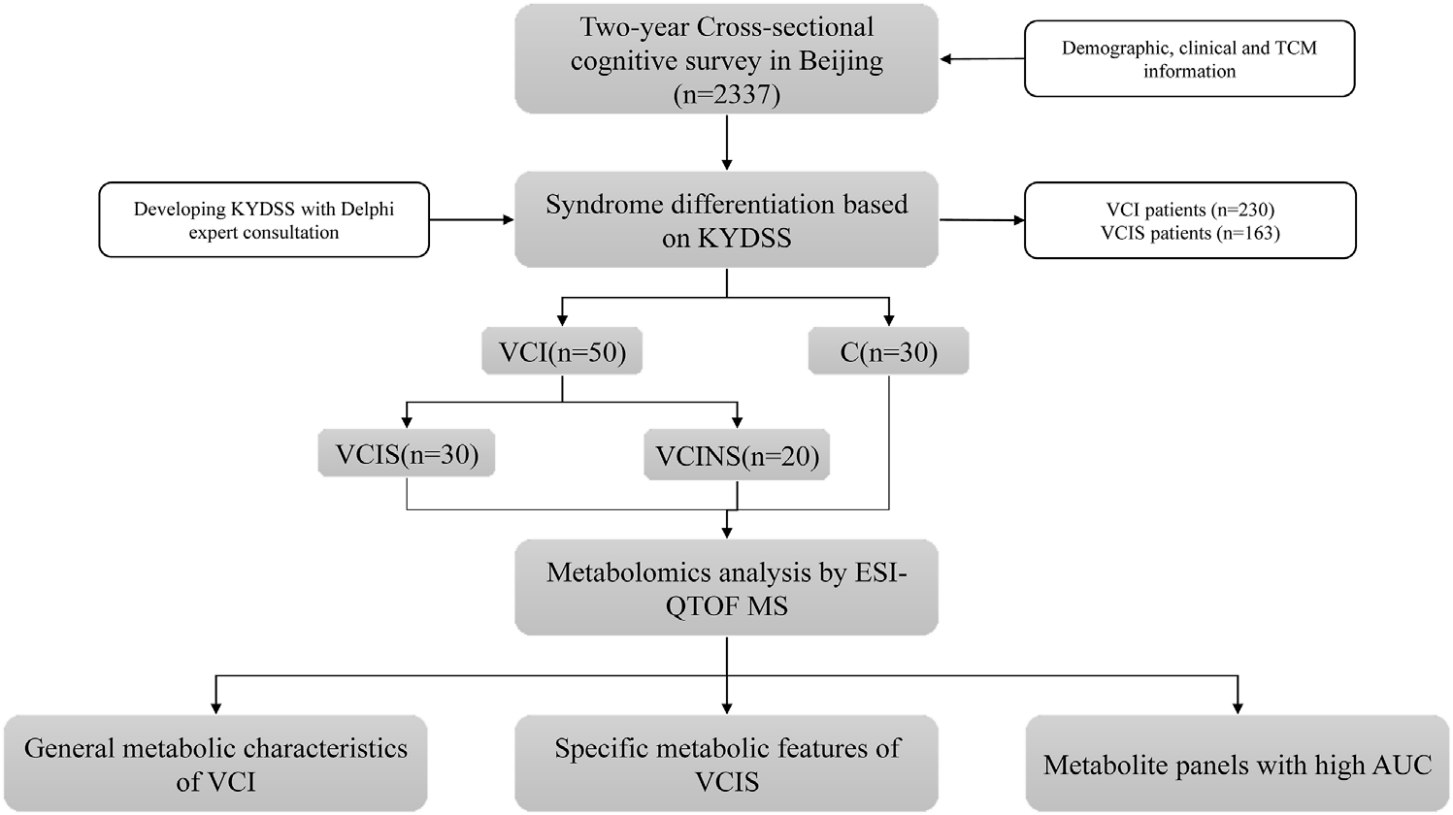
The flowchart of the strategy of the study.

The workflow of the study is illustrated in Figure 1. The general clinical characteristics of VCIS, VCINS, and C are presented in Table 1. There were no significant differences between VCIS and VCINS in terms of age, gender, education, type of work, annual income, AD8 score, working years, frequent physical exercise, the medical history of CAD, hypertension, dyslipidemia, diabetes, FAVB12, as well as smoking and drinking history. There were no significant differences between the groups in terms of BMI, DBP, FPG, HbA1c, TG, TC, LDL-C, and HDL-C. VCIS was associated with a higher MoCA score, KYDS score, depression score, SBP and Hcy, with a greater incidence of nocturnal urination. Marital status and living habits are shown in Supplementary Table S1. In the present study, we also investigated the association between cognitive scales, KYDSS and age. Our analyses revealed a positive correlation between AD8S and KYDSS, while MoCA exhibited a negative correlation with both AD8S and KYDSS. In addition, there was a positive correlation between age and both AD8S and KYDSS; however, there was a negative correlation between age and MoCA Supplementary Table S2. The cognition of different TCM syndromes was represented in Supplementary Table S3. In order to provide more detailed baseline information, we grouped the groups by gender again, and the detailed information is displayed in Supplementary Table S4 and Supplementary Table S5.

**TABLE 1:** The general clinical characteristics of VCIS, VCINS, and C.

	VCIS (n = 30)	VCINS (n = 20)	C (n = 30)	*p* Values
Age (years)	75.07 ± 8.06	70.50 ± 10.70	54.97 ± 7.40	<0.01^*#^
male, n (%)	12 (40.0)	8 (40.0)	6 (20.0)	0.18
Education (%)				0.15
low	7 (23.3)	2 (10.0)	6 (20.0)	
intermediate	16 (53.3)	13 (65.0)	10 (33.3)	
high	7 (23.3)	5 (25.0)	14 (46.7)	
Type of work				0.75
physical labor	11 (36.7)	5 (25.0)	9 (30.0)	
mental labor	9 (30.0)	9 (45.0)	9 (30.0)	
both	10 (33.3)	6 (30.0)	12 (40.0)	
Annual income				0.58
less than 5000RMB	3 (15.0)	9 (30.0)	5 (16.7)	
5,000-20000RMB	7 (35.0)	9 (30.0)	8 (26.7)	
more than 20000RMB	10 (50.0)	12 (40.0)	17 (56.7)	
AD8 score	3.50 ± 1.61	3.90 ± 6.21	0.1 ± 0.31	<0.01^*#^
MOCA score	11.43 ± 4.71	20.15 ± 4.87	30.00 ± 0	<0.01^*#$^
KYDS scale score	25.30 ± 8.67	5.60 ± 1.85	2.93 ± 1.46	<0.01^*$^
Depression score	7.53 ± 6.46	4.00 ± 5.24	1.10 ± 2.11	<0.01^*#$^
Working years	35.38 ± 8.06	36.25 ± 7.91	28.17 ± 7.95	<0.01^*#^
Frequent physical exercise (%)	21 (70.0)	16 (80.0)	26 (86.7)	0.28
Number of nocturnal urination	2.35 ± 1.68	0.85 ± 0.95	0.90 ± 0.67	<0.01^*$^
CAD (%)	10 (33.3)	3 (15.0)	0 (0)	<0.01
Hypertension (%)	18 (60.0)	9 (45.0)	0 (0)	<0.01
Dyslipidemia (%)	24 (80.0)	10 (50.0)	0 (0)	<0.01
Diabetes (%)	12 (40.0)	2 (10.0)	0 (0)	<0.01
FAVB12(%)	8 (26.7)	10 (50.0)	0 (0)	<0.01
Current smokers (%)	6 (20.0)	5 (25.0)	0 (0)	0.02
Current drinkers (%)	6 (20.0)	4 (20.0)	0 (0)	0.03
BMI (kg/m^2^)	24.11 ± 3.22	25.04 ± 3.14	23.67 ± 4.85	0.48
SBP (mmHg)	145.90 ± 24.90	132.10 ± 15.26	127.79 ± 14.07	<0.01^*$^
DBP (mmHg)	79.10 ± 12.68	78.15 ± 11.15	80.18 ± 9.66	0.83
FPG (mmol/L)	6.31 ± 1.90	5.67 ± 1.16	4.93 ± 0.98	<0.01^*^
HbA1c (%)	6.04 ± 1.32	5.69 ± 0.51	5.28 ± 0.55	0.01^*^
TG (mmol/L)	1.46 ± 0.78	1.77 ± 1.25	1.18 ± 0.61	0.08
TC (mmol/L)	4.35 ± 1.00	4.13 ± 0.90	4.50 ± 1.21	0.51
LDL-C (mmol/L)	2.34 ± 0.77	2.24 ± 0.58	2.69 ± 0.86	0.14
HDL-C (mmol/L)	1.53 ± 0.33	1.36 ± 0.44	1.47 ± 0.38	0.32
Hcy (μmol/L)	17.83 ± 7.10	12.06 ± 3.04	15.03 ± 5.07	<0.01^$^

Education: Low educational level included illiteracy and primary school education; Intermediate educational level included middle school education; High educational level included education at post-secondary education, college level or higher. AD8 score.

AD8 Dementia Screening Interview score. MOCA, score: Montreal Cognitive Assessment score. KYDS, score.

Kidney-Yang Deficiency Syndrome Scale score; BMI, body mass index; SBP, systolic blood pressure; DBP, diastolic blood pressure; FPG, fasting blood-glucose, HbA1c glycosylated hemoglobin, TG, triglyceride; TC, total cholesterol; LDL-C, low-density lipoprotein; HDL-C, high-density lipoprotein, Hcy homocysteine. *p < 0.05 VCIS, vs. HC. #*p* < 0.05 VCINS, vs. HC. $*p* < 0.05 VCI, vs. VCINS.

### 3.2 Correlation between differential metabolites and scales

**FIGURE 2:**
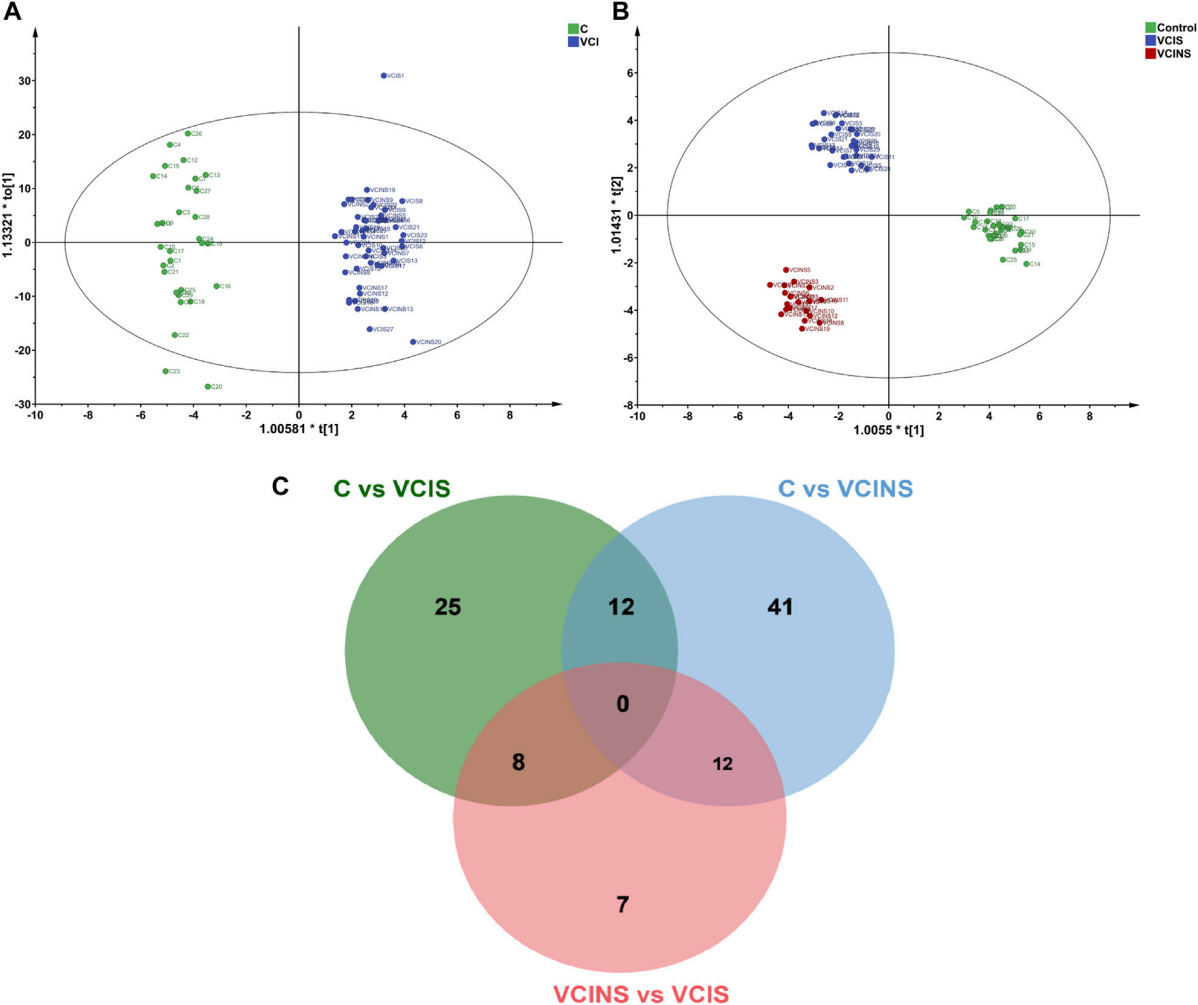
The projection to latent structure-discriminant analysis partial least squares-discriminant analysis (PLS-DA) score plots of VCI, VCIS, VCINS and C groups and venn plot of metabolites. **(A)**: PLS-DA score plot of VCI vs. C **(B)**: PLS-DA score plot of VCIS, VCINS and C groups. **(C)**: Venn plot of differential metabolites.

**FIGURE 3:**
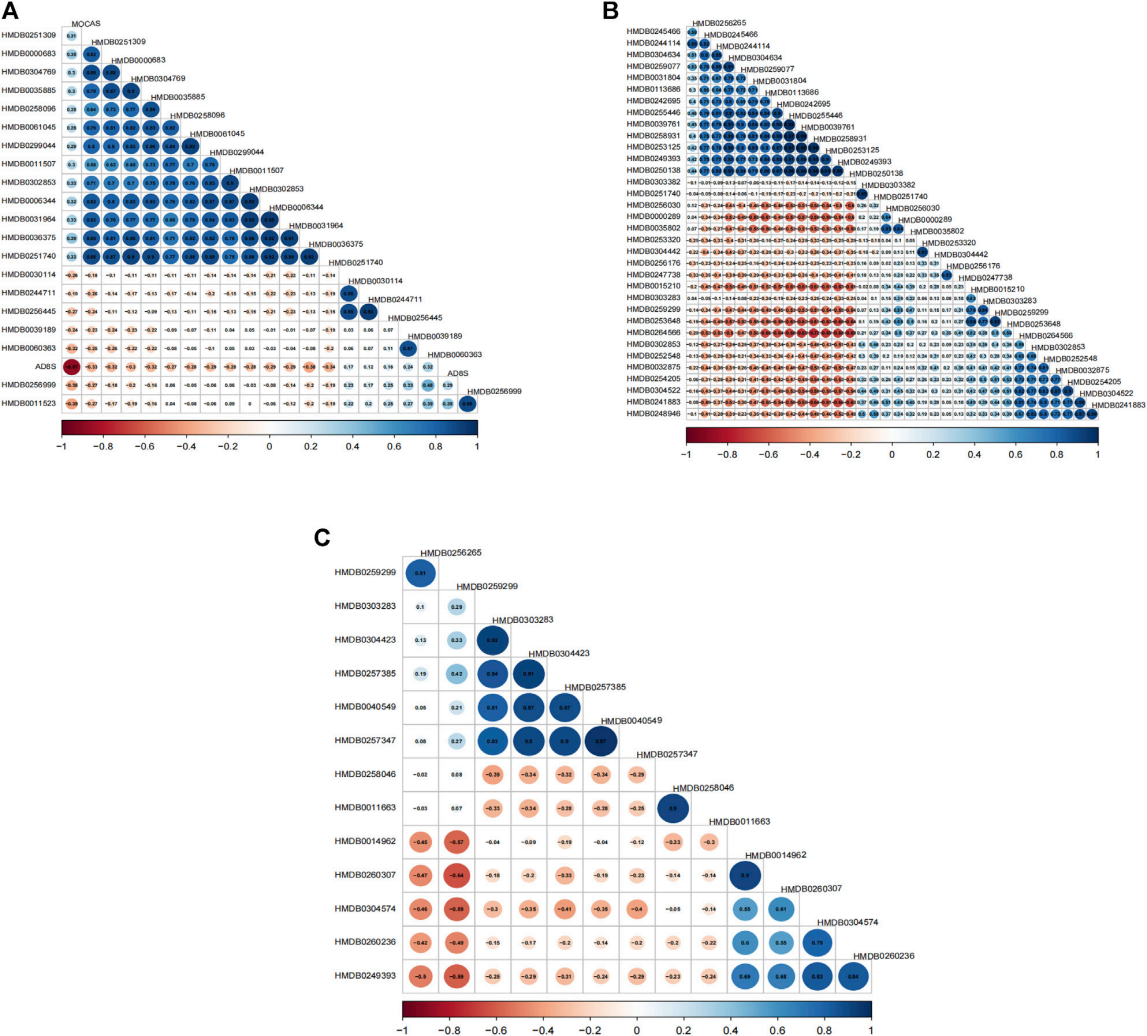
Metabolite correlation diagrams. **(A)**: Correlation diagram of VCIS vs. C **(B)**: Correlation diagram of VCINS vs. C **(C)**: Correlation diagram of VCIS vs. VCINS.

The projection to latent structure-discriminant analysis partial least squares-discriminant analysis (PLS-DA) score plots of groups and venn plot of metabolites were shown in Figure 2. Correlations were calculated between differential metabolites and scales. When comparing the VCIS group with group C, most metabolites exhibited positive correlations with each other, while AD8S showed negative correlations with most metabolites. The highest correlation coefficient (r = 0.95) was observed between (3b,6b,8b, 12a)-8,12-Epoxy-7 (11)-eremophilene-6-angeloyloxy-8,12-dimethoxy-3-ol (HMDB0031964) and phenylacetylglutamine (HMDB0006344) (Figure 3A). When comparing the VCINS group and group C, metabolites exhibited both positive and negative correlations with each other and the highest correlation coefficient (r = 0.99) was observed between 1,1'-[1,13-Tridecanediylbis (oxy)] bisbenzene (HMDB0039761) and 2-Naphthalenesulfonic acid (HMDB0255446) (Figure 3B). Similarly, when comparing the VCIS group with the VCINS group, metabolites also exhibited both positive and negative correlations with each other and the highest correlation coefficient (r = 0.97) was observed between (8S,9S,10R,11S,13S,14S, 17S)-11,17-Dihydroxy-10,13-dimethyl-17-prop-1-ynyl-9,11,12,14,15,16-hexahydro-8H-cyclopenta [a]phenanthren-3-one (HMDB0257347) and xi-2,3-Octadiene-5,7-diyn-1-ol (HMDB0040549) (Figure 3C).

### 3.3 Establishing diagnostic models for VCIS and VCINS

**FIGURE 4:**
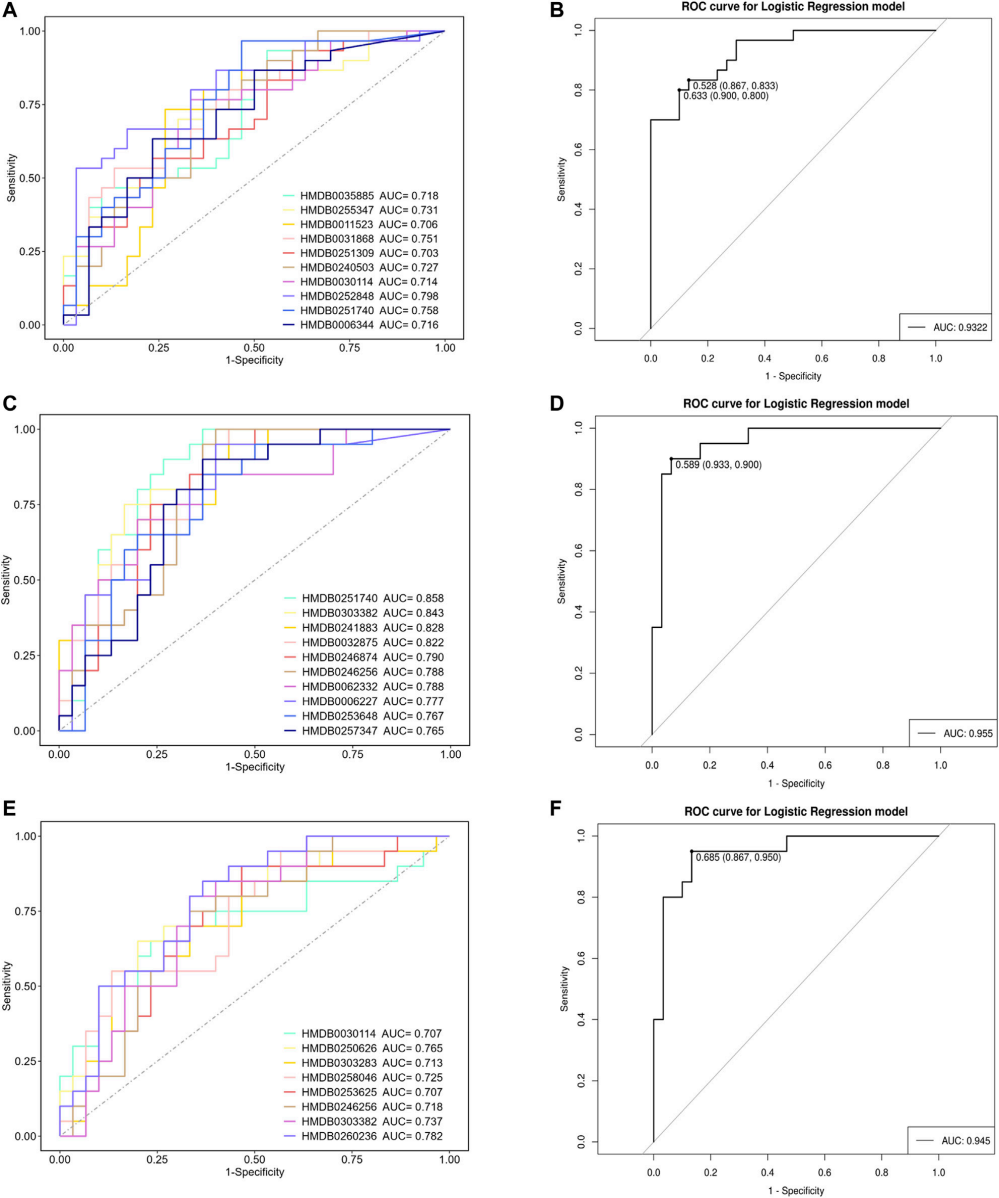
Analysis of receiver operating characteristic (ROC) curves for distinguishing VCIS, VCINS and C **(A)**: ROC curves for distinguishing VCIS from C using metabolites with AUC >0.7. **(B)** Metabolite panel for distinguishing VCIS from C **(C)**: ROC curves for distinguishing VCINS from C using metabolites with AUC >0.7. **(D)** Metabolite panel for distinguishing VCINS from C **(E)**: ROC curves for distinguishing VCIS from VCINS using metabolites with AUC >0.7. **(F)** Metabolite panel for distinguishing VCIS from VCINS.

To further validate the potential diagnostic efficacy of differential metabolites, we established diagnostic models based on metabolite comparisons and selected metabolites with an AUC >0.7 to form a diagnostic panel. Glycerophospho-N-Arachidonoyl Ethanolamine (HMDB0252848) exhibited the highest diagnostic efficacy (AUC = 0.798) when comparing the VCIS group and group C (Figure 4A), and a metabolite panel consisting of 10 metabolites achieved an AUC of 0.9322 (Figure 4B). Eldecalcitol (HMDB0251740) showed the highest diagnostic efficacy (AUC = 0.858) when comparing the VCINS group and group C (Figure 4C), and the AUC of a metabolite panel consisting of 10 metabolites was 0.955 (Figure 4D). 1-(7Z-Hexadecenoyl)-2-(4Z,7Z,10Z,13Z,16Z, 19Z-docosahexaenoyl)-sn-glycero-3-phosphocholine (HMDB0260236) exhibited the highest diagnostic efficacy (AUC = 0.782) when comparing the VCIS group and the VCINS group (Figure 4E), and a metabolite panel consisting of 8 metabolites achieved an AUC of 0.945 (Figure 4F).

### 3.4 Changes in the levels of PE and lysoPE in VCIS were related to an increase in PLA2 levels

**FIGURE 5:**
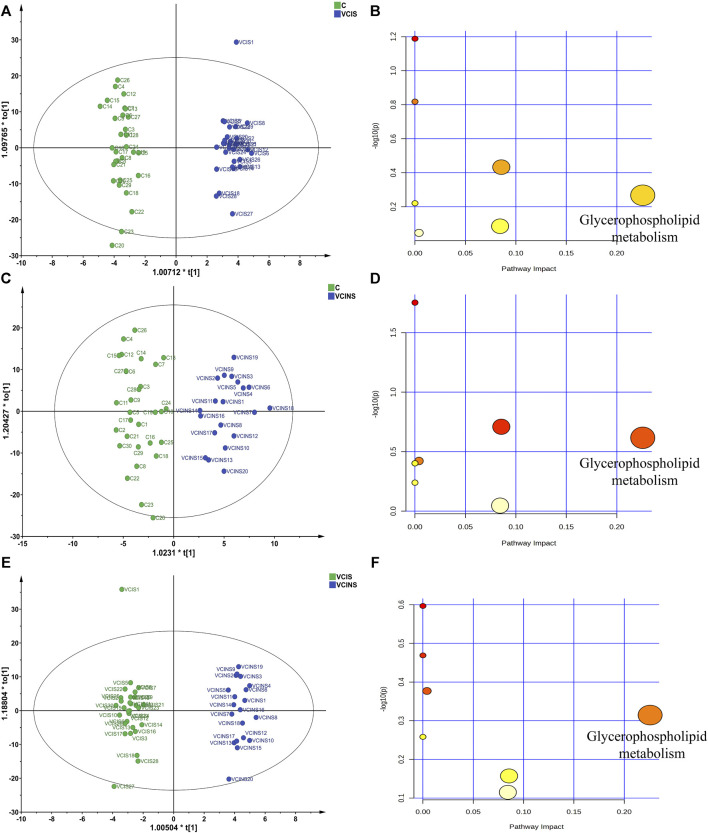
The partial least squares-discriminant analysis (PLS-DA) score plots of pairwise comparison models and pathway enrichment analysis of discriminated metabolites. **(A)**: PLS-DA score plots of VCIS vs. C **(B)**: Pathway impact of VCIS vs. C **(C)**: PLS-DA score plots of VCINS vs. C **(D)**: Pathway impact of VCINS vs. C **(E)**: PLS-DA score plots of VCIS vs. VCINS. **(F)**: Pathway impact of VCIS vs. VCINS.

**FIGURE 6:**
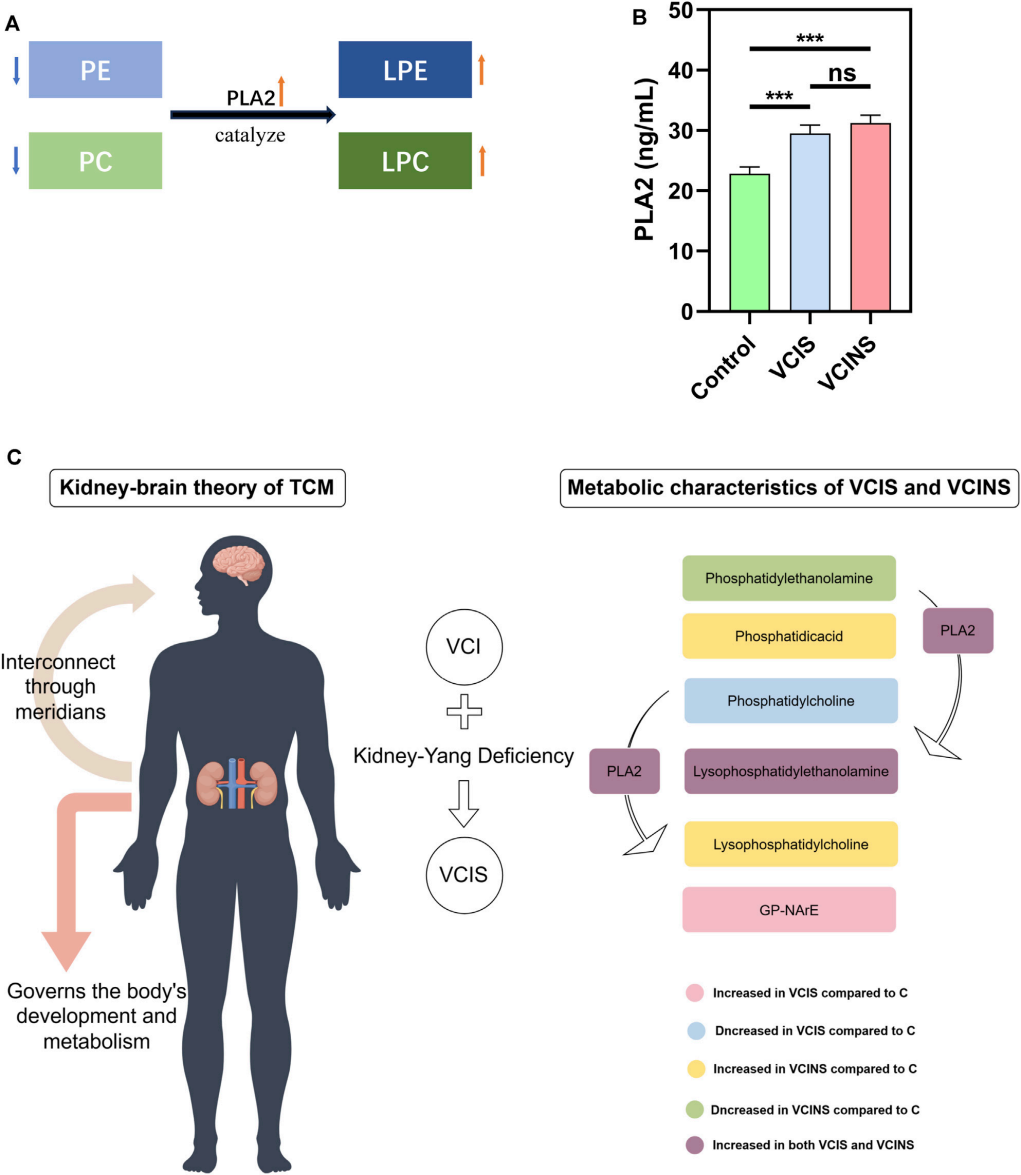
Trend in Phospholipase A2 (PLA2) enzyme activity and the potential metabolic alterations in VCIS and VCINS. **(A)**: Role of PLA2 enzyme in transformation of PE, PC LPE and LPC. **(B)**: Content of PLA2 enzyme in the serum of three groups. **(C)**: The Kidney-brain theory of TCM and the metabolic alterations of VCI (including VCIS and VCINS) patients.

Based on the large proportion of glycerophospholipids in differential metabolites and because pathway enrichment was most related to glycerophospholipid metabolism (Figure 5), we used an ELISA kit to detect the levels of PLA2, as a key enzyme associated with PE, LPE, PC and LPC conversion (Figure 6A). Analysis showed that PLA2 was significantly elevated in both VCIS and VCINS (Figure 6B). Compared to group C, we observed that LPE was upregulated and PC was downregulated in VCIS. Furthermore, in VCINS, LPE and LPC were upregulated and PE was downregulated (Table 2). Therefore, the observed increase of PLA2 was consistent with the conversion of PE, LPE, PC and LPC.

**TABLE 2:** Representative differential metabolites of 3 comparisons.

	Metabolites	m/z	RT (min)	*p*-value	VIP	FC
VCIS vs. C	Glycerophospho-N-Arachidonoyl Ethanolamine	500.28	6.03	0.0001	2.56	1.60
	LysoPE (22:6)	527.27	6.00	0.025	1.43	1.56
	LysoPE (18:2)	478.29	5.99	0.012	1.59	1.28
	LysoPE (22:4)	528.31	6.12	0.019	1.55	1.40
	PC (18:1-O/14:0)	746.53	5.32	0.013	1.45	0.72
	LysoPE (18:0)	480.31	7.69	0.041	1.44	1.34
	LysoPE (18:1)	478.29	6.79	0.025	1.43	1.50
	PC (20:5-OH/P-18:1)	806.57	9.43	0.032	1.30	0.71
VCINS vs. C	PA (19:2/18:1-2OH)	745.51	9.83	0.009	1.79	1.61
	PE (P-18:0/PGF1alpha)	802.56	8.99	0.025	1.30	0.59
	PE-NMe(22:6/20:1)	832.58	10.54	0.040	1.30	0.67
	LysoPC (16:1)	494.32	5.61	0.047	1.28	1.32
	LysoPE (18:1)	478.29	6.79	0.028	1.15	1.68
	Eldecalcitol	489.36	7.72	0.0002	2.08	2.55
VCINS vs. VCIS	1-(Hexadecenoyl)-2-(docosahexaenoyl)-sn-glycero-3-phosphocholine	804.55	9.15	0.002	1.87	1.77
	Undeca-3,6-dienoylcarnitine	326.23	5.32	0.035	1.64	1.54
	H-D-Val-Leu-Lys-pNA	479.30	5.32	0.034	1.63	1.53
	Isocoumarin	147.04	1.38	0.025	1.58	1.26

## 4 Discussion

By carrying out a 2-year cross-sectional cognitive survey, we were able to substantiate the most prominent TCM syndrome associated with VCI. In the survey, involving 2,337 participants, we found that the prevalence of VCI was 9.84% and the prevalence of KYDS was 43.88%; VCIS accounted for 70.87% of all VCI cases. Furthermore, KYDS patients were associated with a worse cognitive status Supplementary Table S3. Therefore, VCIS was chosen as the primary study syndrome, while VCINS and C were set as the control groups. Based on baseline controls for known risk factors for VCI (including age, sex, education, smoking, physical activity, BMI, hypertension, chronic hyperglycemia, diabetes mellitus, dyslipidemia and low sodium intake) (Dichgans and Leys, 2017; Van Der Flier et al., 2018), we screened 80 samples for metabolomic detection. It is worth mentioning that due to the obvious correlation between VCI and Kidney-Yang Deficiency, the number of typical VCINS cases was small; therefore, fewer VCINS patients were included than the other two groups.

When the baseline was consistent, the MoCA score in VCIS was still lower than that for VCINS; in addition, depression scores were higher (Table 1); these data showed that cognitive impairment was more severe in VCIS and VCIS patients and that this was accompanied by poor psychosocial functionality (Rock et al., 2014). Furthermore, VCIS patients had a higher incidence of nocturnal urination than patients in the VCINS and C groups (Table 1). TCM theory states that the kidneys regulate fluid metabolism and govern bladder function. When Kidney-Yang is insufficient, fluid accumulation leads to a greater incidence of nocturnal urination. Higher levels of SBP and Hcy further suggest that the poor cognitive status might be accompanied by vascular injury (Shang et al., 2016; Kim et al., 2019). In addition, our study revealed that cognitive decline and increased Kidney-Yang Deficiency were associated with age progression in patients with VCIS. Furthermore, the severity of cognitive impairment was found to be directly proportional to the degree of Kidney-Yang Deficiency in VCIS patients; notably, this distinctive pattern was not observed in the other two groups, thus highlighting significant clinical characteristics that are unique to VCIS patients Supplementary Table S2.

First, we demonstrated the general metabolic profile of VCI (VCIS and VCINS) patients. Individual metabolite levels from all study participants and HMDB IDs of metabolites have been included in Supplementary Table. A PLS-DA score plot showed a distinct separation between the VCI and C groups (Figure 2A). Furthermore, 45 metabolites were identified between the VCIS and C groups, as well as 65 metabolites that showed significant differences between the VCINS and C groups (Figure 2C). In addition, correlation analysis revealed both positive and negative associations between the majority of these metabolites (Figure 3A, B). Collectively, these results were produced by a stable analytical process and not by over-fitted models Supplementary Figure S1, 2. It is noteworthy that VCI patients exhibited elevated levels of lysophosphatidylethanolamine (LPE), phosphatidic acid (PA), lysophosphatidylcholine (LPC), along with decreased levels of phosphatidylethanolamine (PE), phosphatidylcholine (PC) compared to group C (Table 2). Disorders of these lipids have been associated with cell membrane dysfunction. PA is a glycerophospholipid in which a phosphate moiety occupies a glycerol substitution site. PE is the key regulator of cell membrane fluidity and affects the diffusion of membrane components from lipids to proteins and the dynamics and functionality of integral membrane proteins (Dawaliby et al., 2016). PC may provide a significant supply of choline and maintains structural integrity of the neuronal membrane (Zamroziewicz et al., 2016). As the key factor of lipid mediator production, PLA2 hydrolyses the sn-2 acyl bond of PC to release fatty acids and LPC (Wang et al., 2023), and also catalyzes PE to LPE (Zhu et al., 2022) (Figure 6A). Interestingly, we observed a significant increase in PLA2 expression in both VCIS and VCINS patients (Figure 6B). This upregulation of PLA2 provides an explanation for the dysregulated levels of these metabolites observed in VCI patients. Therefore, the downregulation of PE, and PC, coupled with the upregulation of LP, LPC, PA and increased PLA2 activity, can be considered as general metabolic characteristics associated with VCI. This trend partially aligned with VD patients (Liu et al., 2020).

Secondly, the metabolic characteristics specific to VCIS were investigated by conducting metabolomics assays. VCIS, VCINS and C were separated from each other (Figure 2B), thus indicating significant differences between the three groups. These differences confirmed the feasibility of classification based on clinical scales, thus demonstrating the reliability of KYDSS. Furthermore, a total of 45 differential metabolites were identified in the comparison between VCIS and C, while 27 differential metabolites were identified in the comparison between VCIS and VCINS (Figure 2C). We observed both positive and negative associations among the majority of these metabolites (Figure 3C) In the above comparison, major metabolic disorders were lipids and lipid-like molecules, especially glycerophospholipids. Lipids are maintained in a delicate balance that can easily lead to pathology in the brain when this balance is disturbed (Qin et al., 2021). Phospholipid alterations in the brain are known to be associated with progressive neurodegeneration. Excitotoxicity-induced neurodegeneration is known to contribute to the pathogenesis of cognitive dysfunction in individuals with cerebral ischemia; a metabolic characteristic of this condition is the deficiency of PC and an excess of lysophospholipids, including LPE, in the hippocampus (Sabogal-Guáqueta et al., 2018); the same phenomenon was observed in VCIS patients. Furthermore, increased levels of LPE are strongly correlated with pro-inflammatory response (Villamil-Ortiz and Cardona-Gomez, 2015), while inflammation in the central nervous system is closely related to neurodegeneration (Na et al., 2014). Since a multitude of neuropathological processes can lead to the dysfunction of LPE and PC, it is likely that aberrant levels of these phospholipids are linked to neuronal disease. Moreover, LPE is recognized as a useful biomarker for predicting the conversion of MCI to Alzheimer’s disease (AD) (Llano et al., 2021); this might be due to their intimate association.

Notably, we exclusively observed a significantly aberrant metabolite, Glycerophospho-N-Arachidonoyl Ethanolamine (GP-NArE), in the VCIS group when compared to group C. GP-NArE belongs to a class of organic compounds known as the glycero-3-phospho-n-acyl-ethanolamines. GP-NArE serves as a precursor for N-arachidonoylethanolamine (AEA), an endocannabinoid that plays a pivotal role in modulating neurotransmission and facilitating brain development. Consequently, the dysfunction of GP-NArE can influence neurogenesis, cell differentiation, cell migration, neuronal specification and synaptogenesis in an indirect manner (Anavi-Goffer and Mulder, 2009). In addition, the endocannabinoid system has emerged as a major neuromodulatory system that is critically involved in the control of emotional homeostasis and stress responsiveness (Marco and Laviola, 2012). Although it has been identified as a potential biomarker of lung cancer (Chen et al., 2015), to our knowledge, this is the first identification of GP-NArE in patients with VCI; in addition, GP-NArE exhibited the highest diagnostic efficacy (AUC = 0.798) when comparing the VCIS group and group C. The abnormal levels of GP-NArE explained the poorer brain cognition and emotional homeostasis in VCIS patients. Furthermore, it is evident that GP-NArE represents a key biomarker of VCIS. We hypothesize that the specific increase in GP-NArE levels in VCIS may be associated with a pronounced decline in cerebral nerve function; however, further experiments are required to confirm this hypothesis.

Functional pathway analysis showed that glycerophospholipid metabolism was the most altered pathway in VCIS and VCINS (Figures 5B, D, F). Glycerophospholipid metabolism has been identified as a perturbed pathway of Kidney-Yang Deficiency Syndrome (Nan et al., 2016), while glycerophospholipid disorders leads to alterations in membrane permeability, thereby contributing to the neurodegeneration observed in neurological disorders (Farooqui et al., 2000). The dysfunction of glycerophospholipid metabolism is known to be associated with alterations in key enzymes, particularly PLA2. Significantly elevated levels of PLA2 in both VCIS and VCINS (Figure 6B) were observed; this aligns with the trend observed for glycerophospholipids. In addition to its transformative role in glycerophospholipid metabolism, PLA2 is also implicated in cardiovascular and cerebrovascular diseases and serves as a recognized marker of systemic vascular and neuroinflammation, thus contributing to cerebral/cardiovascular risk. PLA2 has been identified as an independent risk factor for cognitive impairment in cerebral small vessel disease (CSVD) (Zhu et al., 2019). In addition, a systematic review focusing on six studies assessed the association between PLA2 and VCI, further confirming that PLA2 also represents a risk factor for VCI (Qin et al, 2021). Furthermore, PLA2 may play a role in receptor signaling and transcriptional pathways that link oxidative events to an inflammatory response that underlies many neurodegenerative diseases (Sun et al., 2010). Finally, it has been demonstrated that PLA2 enhances the production of proinflammatory and proapoptotic mediators and is therefore considered as one of several inflammatory biomarkers linked to the risk of dementia or impaired cognitive function (Darweesh et al., 2018; Stewart et al., 2019). These findings provide evidence to support the dysregulation of glycerophospholipid metabolism as an intrinsic mechanism underlying VCI and Kidney-Yang Deficiency, with the upregulated expression of PLA2 implicated in inducing this dysregulation. Importantly, PLA2-related cerebral inflammation and neurodegenerative changes may also contribute to the pathogenesis of VCI and Kidney-Yang Deficiency.

The number of patients with dementia is expected to rise from 36 million in 2010 to 115 million in 2050, with VCI accounting for approximately 50%–70% of all dementia cases. Furthermore, individuals diagnosed with VCI experience reduced survival rates when compared to those with Alzheimer’s disease (Van Der Flier et al., 2018). The current diagnosis of VCI relies on clinical symptoms and neuropsychological assessments, and is complemented by neuroimaging techniques, which are both time-consuming and costly, thus necessitating the exploration of alternative VCI biomarkers (Qin et al., 2021). In our study, we observed significant differences in the clinical manifestations and metabolic characteristics between VCIS and VCINS, while conventional VCI risk factors failed to distinguish between the two groups. An accurate diagnosis of VCIS and VCINS would make significant contributions to the selection of appropriate treatments in the early stages of disease, thereby reducing mortality and therapeutic expenditure. Previous research also provided support for the highly predictive nature of metabolites in relation to cognitive decline (Zamroziewicz et al., 2016). Therefore, specific metabolites were selected to construct the diagnostic model; metabolites with an AUC >0.7 were selected to form a metabolite panel in each comparison. As previously stated, GP-NArE exhibited the highest diagnostic efficacy (AUC = 0.798) when comparing the VCIS group and group C (Figure 4A), and the metabolite panel composed of 10 metabolites achieved an AUC of 0.9322 (Figure 4B). Eldecalcitol showed the highest diagnostic efficacy (AUC = 0.858) when the VCINS group was compared with group C (Figure 4C); the AUC of a metabolite panel composed of 10 metabolites as0.955 (Figure 4D). Moreover, 1-(Hexadecenoyl)-2-(docosahexaenoyl)-sn-glycero-3-phosphocholine exhibited superior diagnostic efficacy with an AUC value of 0.782 when differentiating VCIS from VCINS (Figure 4E); a metabolite panel composed of 8 metabolites achieved an AUC of 0.945 (Figure 4F). These findings collectively demonstrated that combining differential metabolites can generate panels with substantial diagnostic potential for distinguishing between VCIS, VCINS, and C, thus enhancing objectivity in VCI diagnosis and providing a foundation for the exploration of future biomarkers.

To sum up, this study had several strengths. First, the accuracy of the study was enhanced by matching the VCIS and VCINS subjects according to age, sex, education, smoking, physical activity, BMI, hypertension, chronic hyperglycemia, diabetes mellitus, dyslipidemia and low sodium intake, which have all been reported to be associated with cognitive function. By integrating clinical cognitive information and TCM syndrome, we confirmed a strong correlation between VCI and Kidney-Yang Deficiency. Furthermore, it was observed that individuals with VCIS exhibited more severe cognitive impairments, poorer psychosocial functioning, increased nocturnal urination frequency, as well as higher levels of SBP and Hcy. We confirmed that the downregulation of PE, and PC coupled with the upregulation of LPE, LPC, PA and PLA2 can be considered as general metabolic characteristics associated with VCI. Secondly, the dysfunction of glycerophospholipids, particularly LPE and PC, was identified as a key metabolic characteristic of VCIS. Notably, GP-NArE was discovered for the first time in VCI patients and proposed as a potential biomarker for VCIS. The upregulation of PLA2 expression was implicated in inducing alterations in glycerophospholipid metabolism in both VCIS and VCINS. Moreover, robust diagnostic models were established based on the detection of metabolites, achieving high AUC values of 0.9322, 0.9550, and 0.9450 respectively. Finally, by integrating insights from both Chinese and Western medicine of VCI, a refined subclassification of VCI disease status was achieved (Figure 6C).

It is important to note that this preliminary description of metabolic characteristics of VCIS was based on a small sample size and limited geographic scope. Therefore, future research will involve conducting a multi-center experiment with larger samples to validate these findings. Furthermore, targeted validation of the aforementioned metabolites will be pursued in subsequent investigations. Finally, based on the preceding cross-sectional findings, we meticulously selected the most representative and noteworthy VCIS for metabolomics investigation, establishing a VCINS group and a C group as comparative benchmarks to offer insights into the objective study of VCI and TCM syndromes. Nevertheless, it should be noted that the TCM syndrome types associated with VCI encompass additional variations that may exhibit distinct metabolic profiles. This aspect warrants further exploration in future investigations.

## 5 Conclusion

Collectively, this study elucidated the metabolic characteristics of VCI patients, investigated the underlying pathogenesis of VCIS and established robust diagnostic metabolite panels to effectively distinguish between VCIS, VCINS, and C. Thus, our findings contribute valuable information relating to the intricate relationship between metabolic disorders in VCI and neurodegeneration as well as vascular/neuroinflammation. Moreover, our findings provide a TCM perspective for the precise diagnosis and treatment of VCI in the context of precision medicine.

## Data Availability

The original contributions presented in the study are included in the article/Supplementary material, further inquiries can be directed to the corresponding author.

## References

[B1] Anavi‐Goffer S. Mulder J. (2009). The polarised life of the endocannabinoid system in CNS development. Chembiochem 10, 1591–1598. 10.1002/cbic.200800827 19533710

[B2] Chen L. Lu W. Wang L. Xing X. Chen Z. Teng X. (2021). Metabolite discovery through global annotation of untargeted metabolomics data. Nat. Meth 18, 1377–1385. 10.1038/s41592-021-01303-3 PMC8733904 34711973

[B3] Chen R. Wang J. Liao C. Zhang L. Guo Q. Wang X. (2018). Exploring the biomarkers and therapeutic mechanism of kidney-yang deficiency syndrome treated by You-gui pill using systems pharmacology and serum metabonomics. RSC Adv. 8, 1098–1115. 10.1039/c7ra12451a 35539000 PMC9077015

[B4] Chen Y. Ma Z. Li A. Li H. Wang B. Zhong J. (2015). Metabolomic profiling of human serum in lung cancer patients using liquid chromatography/hybrid quadrupole time-of-flight mass spectrometry and gas chromatography/mass spectrometry. J. Cancer Res. Clin. Oncol. 141, 705–718. 10.1007/s00432-014-1846-5 25293627 PMC11823701

[B5] Cognitive Impairment Professional Committee NB, Chinese Medical Doctor Association (2019). Guidelines for diagnosis and treatment of vascular cognitive impairment in China 2019. Natl. Med. J. China 99, 2737–2744. 10.3760/cma.j.issn.0376-2491.2019.35.005

[B6] Darweesh S. K. Wolters F. J. Ikram M. A. de Wolf F. Bos D. Hofman A. (2018). Inflammatory markers and the risk of dementia and Alzheimer's disease: a meta-analysis. Alzheimer's dementia 14, 1450–1459. 10.1016/j.jalz.2018.02.014 29605221

[B7] Dawaliby R. Trubbia C. Delporte C. Noyon C. Ruysschaert J.-M. Van Antwerpen P. (2016). Phosphatidylethanolamine is a key regulator of membrane fluidity in eukaryotic cells. J. Biol. Chem. 291, 3658–3667. 10.1074/jbc.M115.706523 26663081 PMC4751403

[B8] Dichgans M. Leys D. (2017). Vascular cognitive impairment. Circ. Res. 120, 573–591. 10.1161/CIRCRESAHA.116.308426 28154105

[B9] Farooqui A. A. Horrocks L. A. Farooqui T. (2000). Glycerophospholipids in brain: their metabolism, incorporation into membranes, functions, and involvement in neurological disorders. Chem. Phys. Lipids 106, 1–29. 10.1016/s0009-3084(00)00128-6 10878232

[B10] Fu C. Zhang N. L. Chen B.-x. Chen Z. R. Jin X. L. Guo R.-j. (2017). Identification and classification of traditional Chinese medicine syndrome types among senior patients with vascular mild cognitive impairment using latent tree analysis. J. Integr. Med. 15, 186–200. 10.1016/S2095-4964(17)60335-2 28494849

[B11] German J. B. Hammock B. D. Watkins S. M. (2005). Metabolomics: building on a century of biochemistry to guide human health. Metabolomics 1, 3–9. 10.1007/s11306-005-1102-8 16680201 PMC1457093

[B12] Jia J. Zhou A. Wei C. Jia X. Wang F. Li F. (2014). The prevalence of mild cognitive impairment and its etiological subtypes in elderly Chinese. Alzheimer's Dementia 10, 439–447. 10.1016/j.jalz.2013.09.008 24418053

[B13] Jiang M. Lu C. Zhang C. Yang J. Tan Y. Lu A. (2012). Syndrome differentiation in modern research of traditional Chinese medicine. J. Ethnopharmacol. 140, 634–642. 10.1016/j.jep.2012.01.033 22322251

[B14] Kim S. Choi B. Y. Nam J. H. Kim M. K. Oh D. H. Yang Y. J. (2019). Cognitive impairment is associated with elevated serum homocysteine levels among older adults. Eur. J. Nutr. 58, 399–408. 10.1007/s00394-017-1604-y 29322314

[B15] Liu Y. Chan D. K. Thalamuthu A. Wen W. Jiang J. Paradise M. (2020). Plasma lipidomic biomarker analysis reveals distinct lipid changes in vascular dementia. Comput. Struct. Biotechnol. J. 18, 1613–1624. 10.1016/j.csbj.2020.06.001 32670502 PMC7334482

[B16] Llano D. A. Devanarayan V. AsDN I. (2021). Serum phosphatidylethanolamine and lysophosphatidylethanolamine levels differentiate Alzheimer’s disease from controls and predict progression from mild cognitive impairment. J. Alzheimer's Dis. 80, 311–319. 10.3233/JAD-201420 33523012

[B17] Lu X. Xiong Z. Li J. Zheng S. Huo T. Li F. (2011). Metabonomic study on Kidney-Yang Deficiency syndrome and intervention effects of Rhizoma Drynariae extracts in rats using ultra performance liquid chromatography coupled with mass spectrometry. Talanta 83, 700–708. 10.1016/j.talanta.2010.09.026 21147309

[B18] Marco E. M. Laviola G. (2012). The endocannabinoid system in the regulation of emotions throughout lifespan: a discussion on therapeutic perspectives. J. Psychopharmacol. 26, 150–163. 10.1177/0269881111408459 21693551

[B19] Na K.-S. Jung H.-Y. Kim Y.-K. (2014). The role of pro-inflammatory cytokines in the neuroinflammation and neurogenesis of schizophrenia. Prog. Neuropsychopharmacol. Biol. Psychiatry 48, 277–286. 10.1016/j.pnpbp.2012.10.022 23123365

[B20] Nan Y. Zhou X. Liu Q. Zhang A. Guan Y. Lin S. (2016). Serum metabolomics strategy for understanding pharmacological effects of ShenQi pill acting on kidney yang deficiency syndrome. J. Chromatogr. B 1026, 217–226. 10.1016/j.jchromb.2015.12.004 26747643

[B21] Qin Q. Yin Y. Xing Y. Wang X. Wang Y. Wang F. (2021). Lipid metabolism in the development and progression of vascular cognitive impairment: a systematic review. Front. Neurology 12, 709134. 10.3389/fneur.2021.709134 PMC8639494 34867708

[B22] Rinschen M. M. Ivanisevic J. Giera M. Siuzdak G. (2019). Identification of bioactive metabolites using activity metabolomics. Nat. Rev. Mol. Cell Biol. 20, 353–367. 10.1038/s41580-019-0108-4 30814649 PMC6613555

[B23] Rock P. L. Roiser J. P. Riedel W. J. Blackwell A. (2014). Cognitive impairment in depression: a systematic review and meta-analysis. Psychol. Med. 44, 2029–2040. 10.1017/S0033291713002535 24168753

[B24] Sabogal-Guáqueta A. M. Posada-Duque R. Cortes N. C. Arias-Londoño J. D. Cardona-Gómez G. P. (2018). Changes in the hippocampal and peripheral phospholipid profiles are associated with neurodegeneration hallmarks in a long-term global cerebral ischemia model: attenuation by Linalool. Neuropharmacology 135, 555–571. 10.1016/j.neuropharm.2018.04.015 29680773

[B25] Shang S. Li P. Deng M. Jiang Y. Chen C. Qu Q. (2016). The age-dependent relationship between blood pressure and cognitive impairment: a cross-sectional study in a rural area of Xi'an, China. PLoS ONE 11, e0159485. 10.1371/journal.pone.0159485 27438476 PMC4954703

[B26] Skrobot O. A. Black S. E. Chen C. DeCarli C. Erkinjuntti T. Ford G. A. (2018). Progress toward standardized diagnosis of vascular cognitive impairment: Guidelines from the vascular impairment of cognition classification Consensus study. Alzheimer's Dementia 14, 280–292. 10.1016/j.jalz.2017.09.007 29055812

[B27] Stewart R. A. Held C. Krug‐Gourley S. Waterworth D. Stebbins A. Chiswell K. (2019). Cardiovascular and lifestyle risk factors and cognitive function in patients with stable coronary heart disease. J. Am. Heart Assoc. 8, e010641. 10.1161/JAHA.118.010641 30897999 PMC6509727

[B28] Sun G. Y. Shelat P. B. Jensen M. B. He Y. Sun A. Y. Simonyi A. (2010). Phospholipases A2 and inflammatory responses in the central nervous system. NeuroMol Med. 12, 133–148. 10.1007/s12017-009-8092-z PMC3075861 19855947

[B29] Van Der Flier W. M. Skoog I. Schneider J. A. Pantoni L. Mok V. Chen C. L. (2018). Vascular cognitive impairment. Nat. Rev. Dis. Prim. 4, 18003–18016. 10.1038/nrdp.2018.3 29446769

[B30] Villamil-Ortiz J. G. Cardona-Gomez G. P. (2015). Comparative analysis of autophagy and tauopathy related markers in cerebral ischemia and Alzheimer’s disease animal models. Front. aging Neurosci. 7, 84. 10.3389/fnagi.2015.00084 26042033 PMC4436888

[B31] Wang Y.-n. Zhang Z.-h. Liu H.-j. Guo Z.-y. Zou L. Zhang Y.-m. (2023). Integrative phosphatidylcholine metabolism through phospholipase A2 in rats with chronic kidney disease. Acta Pharmacol. Sin. 44, 393–405. 10.1038/s41401-022-00947-x 35922553 PMC9889763

[B32] Zamroziewicz M. K. Zwilling C. E. Barbey A. K. (2016). Inferior prefrontal cortex mediates the relationship between phosphatidylcholine and executive functions in healthy, older adults. Front. aging Neurosci. 8, 226. 10.3389/fnagi.2016.00226 27733825 PMC5040143

[B33] Zhu Q. Wu Y. Mai J. Guo G. Meng J. Fang X. (2022). Comprehensive metabolic profiling of inflammation indicated key roles of glycerophospholipid and arginine metabolism in coronary artery disease. Front. Immunol. 13, 829425. 10.3389/fimmu.2022.829425 35371012 PMC8965586

[B34] Zhu S. Wei X. Yang X. Huang Z. Chang Z. Xie F. (2019). Plasma lipoprotein-associated phospholipase A2 and superoxide dismutase are independent predicators of cognitive impairment in cerebral small vessel disease patients: diagnosis and assessment. Aging Dis. 10, 834–846. 10.14336/AD.2019.0304 31440388 PMC6675532

