# Supplementary material for: A serum metabolomics study of vascular cognitive impairment patients based on Traditional Chinese medicine syndrome differentiation
Source: Front Mol Biosci. 2023 Dec 5;10:1305439. doi: 10.3389/fmolb.2023.1305439 (PMC10728729; doi:10.3389/fmolb.2023.1305439)
Supplement: Supplementary file 2 [file DataSheet2.docx]

**Kidney-Yang Deficiency Syndrome Scale**

Please answer the following questions based on your feelings and symptoms over the past year.

Criteria for the 1-5 items: 0- Never; 2- Seldom appear; 4- Often appear; 6-Often; 8-Always. Criteria for the 6-10 items: 0- Never; 1- Seldom appear; 2- Often appear; 3-Often; 4-Always.

1. **Lumbago（pain in the lumbar region）**

A. Never B. Seldom appear (a little) C. Often appear D. Often E. Always

1. **Knee weakness and lack of strength (Weak knees/lower limbs, could not stand for a long time).**

A. Never B. Seldom appear (a little) C. Often appear D. Often E. Always

1. **Cold body and limbs (In warm conditions, the body and limbs feel cold).**

A. Never B. Seldom appear (a little) C. Often appear D. Often E. Always

1. **Cold lumbar and knees (The lumbar and knees easily feel cold).**

A. Never B. Seldom appear (a little) C. Often appear D. Often E. Always

1. **Nocturia (More nighttime urination).**

A. Never B. Seldom appear (a little) C. Often appear D. Often E. Always

1. **Tinnitus and deafness (Feeling noise in ear, or varying degrees of hearing loss).**

A. Never B. Seldom appear (a little) C. Often appear D. Often E. Always

1. **Poor libido (Lack of desire for sexual gratification, the same as low libido).**

A. Never B. Seldom appear (a little) C. Often appear D. Often E. Always

1. **Fear of cold (Sensation of cold which can be relieved by warmth, the same as intolerance of cold).**

A. Never B. Seldom appear (a little) C. Often appear D. Often E. Always

1. **Poor tolerance to cold (Can not tolerate cold environments, such as winter, cold air conditioning, or electric fan).**

A. Never B. Seldom appear (a little) C. Often appear D. Often E. Always

1. **Fifth-watch diarrhea (Diarrhea occurring daily at dawn, usually due to kidney yang deficiency, the same as early morning diarrhea).**

A. Never B. Seldom appear (a little) C. Often appear D. Often E. Always

Total score:
