# Supplementary material for: A serum metabolomics study of vascular cognitive impairment patients based on Traditional Chinese medicine syndrome differentiation
Source: Front Mol Biosci. 2023 Dec 5;10:1305439. doi: 10.3389/fmolb.2023.1305439 (PMC10728729; doi:10.3389/fmolb.2023.1305439)
Supplement: Supplementary file 3 [file DataSheet1.docx]

Supplementary Material

# Supplementary Figures and Tables

For more information on Supplementary Material and for details on the different file types accepted, please see [here](https://www.frontiersin.org/guidelines/author-guidelines#supplementary-material).

## Supplementary Figures


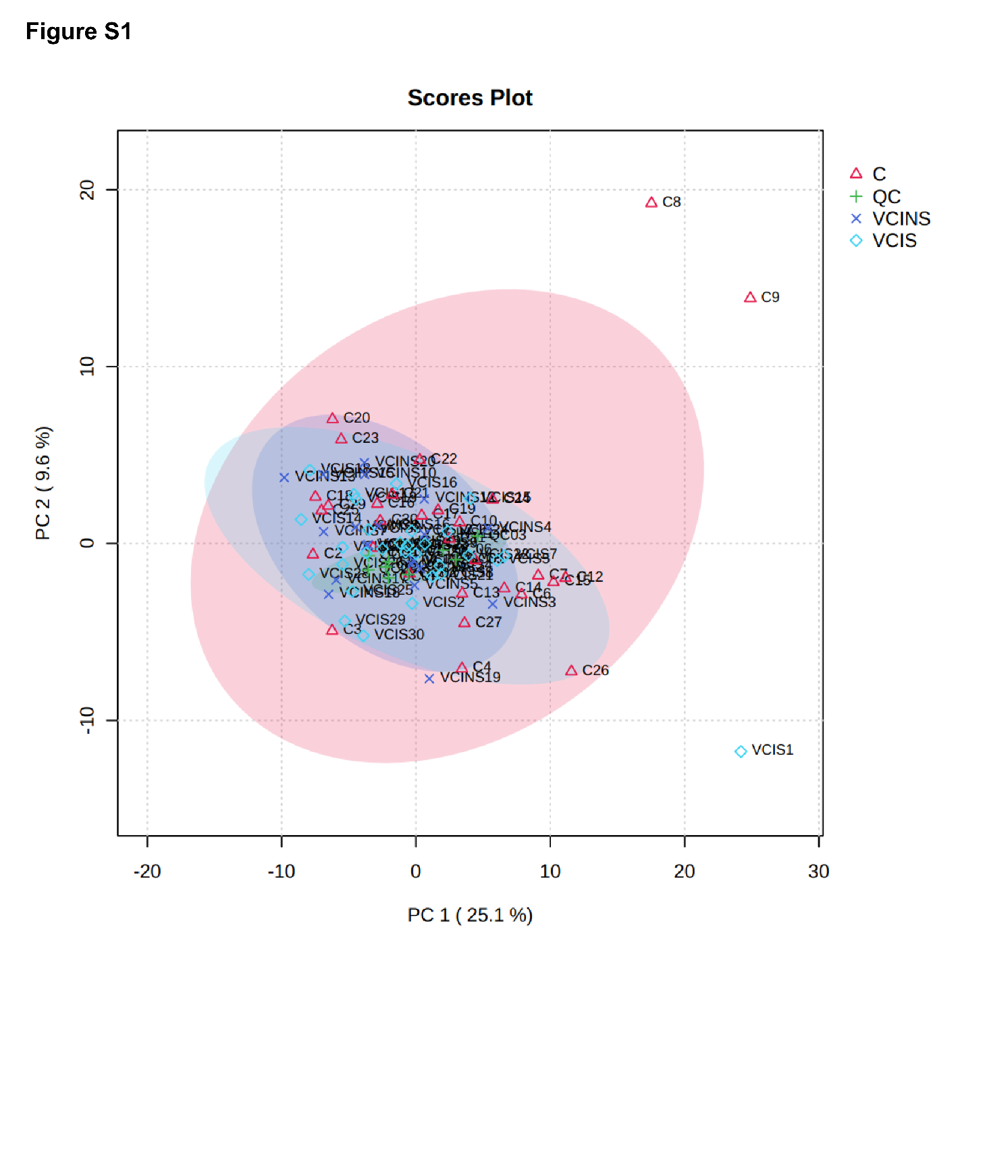


**Supplementary Figure 1.** PLS-DA scores plot of three groups.


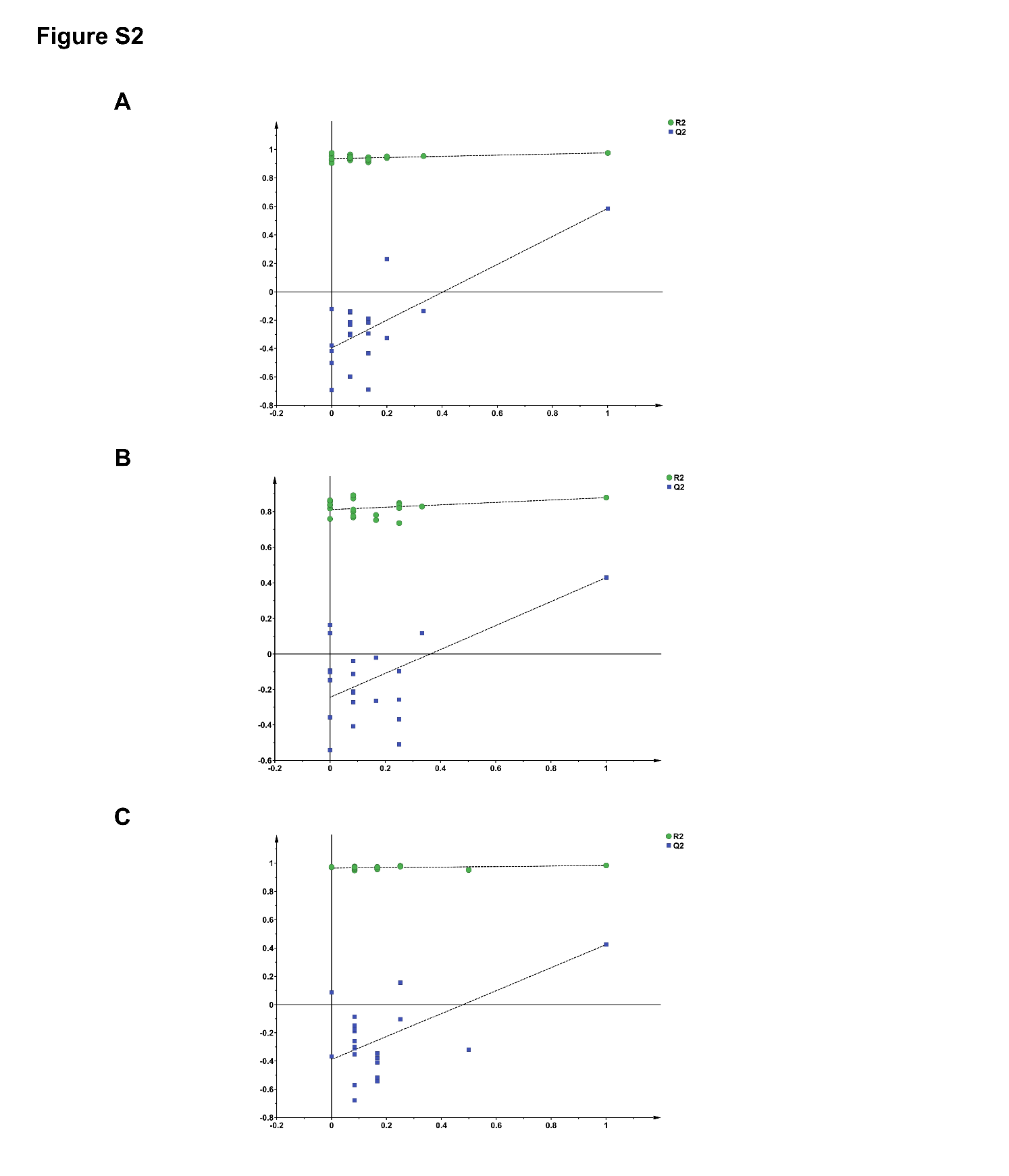


**Supplementary Figure 2.** (A): Permutation of VCIS vs C. (B): Permutation of VCINS vs C. (C): Permutation of VCIS vs VCINS.

## Supplementary Tables

**Supplementary table 1 Marital status and living habits**

|  | | VCIS(n=30) | | VCINS(n=20) | C(n=30) | P values | |  |
| --- | --- | --- | --- | --- | --- | --- | --- | --- |
| Marital status(％) | |  | |  |  | 0.03 | |  |
| married | | 20(66.7) | | 19(95.0) | 26(86.7) |  | |  |
| never married, divorced, widowed | | 10(33.3) | | 1(5.0) | 4(13.3) |  | |  |
| Taste(％) | |  | |  |  | 0.21 | |  |
| salty | | 12(40.0) | | 5(25.0) | 2(6.7) |  | |  |
| pale | | 4(13.3) | | 5(25.0) | 4(13.3) |  | |  |
| moderate | | 14(46.7) | | 10(50.0) | 24(80.0) |  | |  |
| Meat and vegetable preference(％) | |  | |  |  | 0.01 | |  |
| more meat | | 4(13.3) | | 2(10.0) | 4(13.3) |  | |  |
| more vegetable | | 14(46.7) | | 5(25.0) | 2(6.7) |  | |  |
| balance diet | | 12(40.0) | | 13(65.0) | 24(80.0) |  | |  |
| Vegetable(％) | |  | |  |  | 0.25 | |  |
| ≥5 days per week | | 12(40.0) | | 14(70.0) | 17(56.7) |  | |  |
| 3-4 days per week | | 13(43.3) | | 5(25.0) | 11(36.7) |  | |  |
| ≤2 days per week | | 5(16.7) | | 1(5.0) | 2(6.7) |  | |  |
| Fecal texture (％) |  | |  | |  | | 0.96 | |
| sloppy stool | 3(10.0) | | 2(10.0) | | 0(0) | |  | |
| normal stool | 18(60.0) | | 14(70.0) | | 27(90.0) | |  | |
| dry stool | 9(30.0) | | 4(20.0) | | 3(10.0) | |  | |
| Urine |  | |  | |  | | 0.77 | |
| yellow, reddish urine | 1(3.3) | | 1(5.0) | | 1(3.3) | |  | |
| Normal | 28(93.3) | | 19(95.0) | | 29(96.7) | |  | |
| clear urine in large amounts | 1(3.3) | | 0(0) | | 0(0) | |  | |

*P < 0.05 VCIS vs. HC. #P < 0.05 VCINS vs. HC. $P < 0.05 VCI vs. VCINS.

**Supplementary table 2 The correlation between clinical scales in VCIS patients**

| Scale | | r | P values |
| --- | --- | --- | --- |
| AD8S | MoCA | -0.87 | ＜0.05 |
| AD8S | KYDSS | 0.75 | ＜0.05 |
| AD8S | Age | 0.73 | ＜0.05 |
| MoCA | KYDSS | -0.80 | ＜0.05 |
| MoCA | Age | -0.78 | ＜0.05 |
| KYDSS | Age | 0.70 | ＜0.05 |

**Supplementary table 3 The cognition of different TCM syndromes**

| Scale | Non-KYDS | KYDS | P values |
| --- | --- | --- | --- |
| HAMD | 2.00±2.59 | 4.94±4.37 | ＜0.01 |
| AD8 | 0.25±1.28 | 0.79±1.27 | ＜0.01 |
| MOCA | 29.63±2.10 | 28.17±4.69 | ＜0.01 |

**Supplementary table 4 The general clinical characteristics of VCIS, VCINS, and C(male)**

|  | **VCIS (n = 12)** | **VCINS (n = 8)** | **C (n = 6)** | ***P* values** |
| --- | --- | --- | --- | --- |
| Age(years) | 75.58±9.02 | 73.38±9.52 | 57.33±10.25 | 0.02^*#^ |
| Education(％) |  |  |  | 0.62 |
| low | 2(16.7) | 2(25.0) | 1(16.7) |  |
| intermediate | 5(41.7) | 5(62.5) | 2(33.3) |  |
| high | 5(41.7) | 1(12.5) | 3(50.0) |  |
| Type of work |  |  |  | 0.28 |
| physical labor | 5(41.7) | 2(25.0) | 3(50.0) |  |
| mental labor | 5(41.7) | 4(50.0) | 0(0) |  |
| both | 2(16.7) | 2(25.0) | 3(50.0) |  |
| Annual income |  |  |  | 0.48 |
| less than 5000RMB | 1(8.3) | 1(12.5) | 1(16.7) |  |
| 5000-20000RMB | 2(16.7) | 3(37.5) | 0(0) |  |
| more than 20000RMB | 9(75.0) | 4(50.0) | 5(83.3) |  |
| AD8 score | 3.58±1.98 | 2.75±1.17 | 0.00±0 | < 0.01^*#^ |
| MOCA score | 11.17±4.57 | 20.38±4.31 | 30.00±0 | < 0.01^*#$^ |
| KYDS scale score | 21.67±6.11 | 6.00±1.77 | 2.67±1.21 | < 0.01^*$^ |
| Depression score | 6.08±5.04 | 3.25±4.62 | 0.50±1.23 | 0.05^*^ |
| Working years | 39.75±6.06 | 41.75±3.77 | 29.17±12.59 | 0.01^*#^ |
| Frequent physical exercise(％) | 6(50.0) | 5(62.5) | 4(66.7) | 0.76 |
| Number of nocturnal urination | 2.46±2.25 | 1.00±1.34 | 1.17±0.75 | 0.16 |
| Waistline | 89.92±8.79 | 89.88±9.55 | 101.33±21.71 | 0.19 |
| CAD(％) | 3(25.0) | 1(12.5) | 0(0) | 0.37 |
| Hypertension(％) | 6(50.0) | 4(50.0) | 0(0) | 0.09 |
| Dyslipidemia(％) | 9(75.0) | 4(50.0) | 0(0) | 0.01 |
| Diabetes (%) | 5(41.7) | 0(0) | 0(0) | 0.03 |
| FAVB12(％) | 3(25.0) | 4(50.0) | 0(0) | 0.11 |
| Current smokers(％) | 5(41.7) | 5(62.5) | 0(0) | 0.06 |
| Current drinkers(％) | 4(33.3) | 4(50.0) | 0(0) | 0.13 |
| BMI (kg/m²) | 24.00±3.10 | 26.35±2.84 | 27.11±8.62 | 0.37 |
| SBP (mmHg) | 148.33±28.57 | 136.13±13.40 | 136.83±6.65 | 0.38 |
| DBP (mmHg) | 86.17±13.54 | 81.75±11.89 | 86.33±10.33 | 0.70 |
| FPG (mmol/L) | 6.48±2.17 | 5.48±1.35 | 4.80±0.74 | 0.14 |
| HbA1c(％) | 6.11±1.60 | 5.84±0.62 | 5.53±0.39 | 0.62 |
| TG (mmol/L) | 1.31±0.65 | 1.68±1.23 | 1.03±0.45 | 0.37 |
| TC (mmol/L) | 3.76±0.99 | 3.83±0.89 | 4.58±1.27 | 0.28 |
| LDL-C(mmol/L) | 2.13±0.82 | 2.17±0.54 | 3.12±0.82 | 0.06 |
| HDL-C(mmol/L) | 1.42±0.27 | 1.26±0.46 | 1.25±0.30 | 0.49 |
| Hcy(μmol/L) | 19.78±6.88 | 13.85±2.52 | 16.10±5.16 | 0.07 |

^Education: Low educational level included illiteracy and primary school education; Intermediate educational level included middle school education; High educational level included education at post-secondary education, college level or higher. AD8 score:^ ^AD8 Dementia Screening Interview score. MOCA score: Montreal Cognitive Assessment score. KYDS score:^ ^Kidney-Yang Deficiency Syndrome Scale score; BMI body mass index, SBP systolic blood pressure, DBP diastolic blood pressure, FPG fasting blood-glucose, HbA1c glycosylated hemoglobin, TG triglyceride, TC total cholesterol, LDL-C low-density lipoprotein, HDL-C high-density lipoprotein, Hcy homocysteine. *P < 0.05 VCIS vs. HC. #P < 0.05 VCINS vs. HC. $P < 0.05 VCI vs. VCINS.^

**Supplementary table 5 The general clinical characteristics of VCIS, VCINS, and C(female)**

|  | **VCIS (n = 18)** | **VCINS (n = 12)** | **C (n = 24)** | ***P* values** |
| --- | --- | --- | --- | --- |
| Age(years) | 74.72±7.60 | 68.58±11.41 | 54.38±6.66 | < 0.01^*#^ |
| Education(％) |  |  |  | 0.05 |
| low | 5(27.8) | 0(0) | 5(20.8) |  |
| intermediate | 11(61.1) | 8(66.7) | 8(33.3) |  |
| high | 2(11.1) | 4(33.3) | 11(45.8) |  |
| Type of work |  |  |  | 0.81 |
| physical labor | 6(33.3) | 3(25.0) | 6(25.0) |  |
| mental labor | 4(22.2) | 5(41.7) | 9(37.5) |  |
| both | 8(44.4) | 4(33.3) | 9(37.5) |  |
| Annual income |  |  |  | 0.13 |
| less than 5000RMB | 8(44.4) | 2(16.7) | 4(16.7) |  |
| 5000-20000RMB | 7(38.9) | 4(33.3) | 8(33.3) |  |
| more than 20000RMB | 3(16.7) | 6(50.0) | 12(50.0) |  |
| AD8 score | 3.44±1.38 | 4.67±8.00 | 0.13±0.34 | < 0.01^*#^ |
| MOCA score | 11.61±4.92 | 20.00±5.39 | 30.00±0 | < 0.01^*#$^ |
| KYDS scale score | 27.72±9.41 | 5.33±1.92 | 3.00±1.53 | < 0.01^*$^ |
| Depression score | 8.50±7.22 | 4.50±5.76 | 1.25±2.27 | < 0.01^*#^ |
| Working years | 31.64±7.82 | 32.58±7.82 | 27.81±6.63 | 0.14 |
| Frequent physical exercise(％) | 15(83.3) | 11(91.7) | 22(91.7) | 0.66 |
| Number of nocturnal urination | 2.28±1.24 | 0.75±0.62 | 0.83±0.65 | < 0.01^*$^ |
| Waistline | 86.12±13.04 | 82.42±10.07 | 80.00±7.75 | 0.20 |
| CAD(％) | 7(38.9) | 2(16.7) | 0(0) | < 0.01 |
| Hypertension(％) | 12(66.7) | 5(41.7) | 0(0) | < 0.01 |
| Dyslipidemia(％) | 15(83.3) | 6(50.0) | 0(0) | < 0.01 |
| Diabetes (%) | 7(38.9) | 2(16.7) | 0(0) | < 0.01 |
| FAVB12(％) | 5(27.8) | 6(50.0) | 0(0) | < 0.01 |
| Current smokers(％) | 1(5.6) | 0(0) | 0(0) | 0.36 |
| Current drinkers(％) | 2(11.1) | 0(0) | 0(0) | 0.13 |
| BMI (kg/m²) | 24.19±3.39 | 24.18±3.13 | 22.73±2.88 | 0.27 |
| SBP (mmHg) | 144.18±22.71 | 129.42±16.38 | 125.32±14.63 | < 0.01^*$^ |
| DBP (mmHg) | 74.12±9.57 | 75.75±10.44 | 78.50±9.00 | 0.36 |
| FPG (mmol/L) | 6.19±1.76 | 5.81±1.05 | 4.96±1.05 | 0.02 |
| HbA1c(％) | 5.99±1.15 | 5.58±0.41 | 5.20±0.57 | 0.01* |
| TG (mmol/L) | 1.56±0.86 | 1.83±1.32 | 1.22±0.65 | 0.17 |
| TC (mmol/L) | 4.75±0.82 | 4.35±0.88 | 4.47±1.22 | 0.55 |
| LDL-C(mmol/L) | 2.45±0.74 | 2.29±0.63 | 2.57±0.86 | 0.67 |
| HDL-C(mmol/L) | 1.60±0.36 | 1.44±0.42 | 1.53±0.38 | 0.52 |
| Hcy(μmol/L) | 16.53±7.13 | 10.75±2.78 | 14.74±5.12 | 0.03^$^ |

^Education: Low educational level included illiteracy and primary school education; Intermediate educational level included middle school education; High educational level included education at post-secondary education, college level or higher. AD8 score:^ ^AD8 Dementia Screening Interview score. MOCA score: Montreal Cognitive Assessment score. KYDS score: Kidney-Yang Deficiency Syndrome Scale score; BMI body mass index, SBP systolic blood pressure, DBP diastolic blood pressure, FPG fasting blood-glucose, HbA1c glycosylated hemoglobin, TG triglyceride, TC total cholesterol, LDL-C low-density lipoprotein, HDL-C high-density lipoprotein, Hcy homocysteine. *P < 0.05 VCIS vs. HC. #P < 0.05 VCINS vs. HC. $P < 0.05 VCI vs. VCINS.^
